# Structural dynamics flexibility informs function and evolution at a proteome scale

**DOI:** 10.1111/eva.12052

**Published:** 2013-02-13

**Authors:** Zeynep Nevin Gerek, Sudhir Kumar, Sefika Banu Ozkan

**Affiliations:** 1 Center for Evolutionary Medicine and Informatics, Biodesign Institute, Arizona State UniversityTempe, AZ, USA; 2 Department of Physics, Center for Biological Physics, Bateman Physical Sciences F-Wing, Arizona State UniversityTempe, AZ, USA; 3 School of Life Sciences, Arizona State UniversityTempe, AZ, USA

**Keywords:** elastic network models, functional genomics, single nucleotide variants, structural dynamics

## Abstract

Protein structures are dynamic entities with a myriad of atomic fluctuations, side-chain rotations, and collective domain movements. Although the importance of these dynamics to proper functioning of proteins is emerging in the studies of many protein families, there is a lack of broad evidence for the critical role of protein dynamics in shaping the biological functions of a substantial fraction of residues for a large number of proteins in the human proteome. Here, we propose a novel dynamic flexibility index (*dfi*) to quantify the dynamic properties of individual residues in any protein and use it to assess the importance of protein dynamics in 100 human proteins. Our analyses involving functionally critical positions, disease-associated and putatively neutral population variations, and the rate of interspecific substitutions per residue produce concordant patterns at a proteome scale. They establish that the preservation of dynamic properties of residues in a protein structure is critical for maintaining the protein/biological function. Therefore, structural dynamics needs to become a major component of the analysis of protein function and evolution. Such analyses will be facilitated by the *dfi*, which will also enable the integrative use of structural dynamics with evolutionary conservation in genomic medicine as well as functional genomics investigations.

## Introduction

The first crystal structure was solved in late 1950, which revolutionized our ability to understand mechanisms underlying protein function and the effect of individual residues whose changes are disrupted (Dill and MacCallum 2012). More recent advancements of experimental and computational techniques are making it clear that the proteins are dynamic entities with the signatures of these dynamics encoded in their tertiary structures (Frauenfelder et al. 1979; Frauenfelder et al. 1991; Dill and Chan 1997; James and Tawfik 2003; Eisenmesser et al. 2005; Henzler-Wildman and Kern 2007; Teilum et al. 2009; Kamerlin and Warshel 2010; Villali and Kern 2010). Thus, every protein has the potential to adopt many different conformations in the native state, which has made the classic ‘single structure/single function’ dogma untenable (Dill and Chan 1997; James and Tawfik 2003). It is only through inter-conversion among these conformational states in their native ensemble do proteins have the capacity to *efficiently and effectively* carry out proper functions in living cells (Henzler-Wildman and Kern 2007). This property has been seen in a series of experimental and computational studies, including those demonstrating the importance of protein structural dynamics in allosteric regulation (Eisenmesser et al. 2002; Gunasekaran et al. 2004; Wang et al. 2004; Eisenmesser et al. 2005; Zheng et al. 2007; Tsai et al. 2008; Tzeng and Kalodimos 2011; Kalodimos 2012), ligand recognition (Adzhubei et al. 2010; Liu et al. 2010), electron transfer (Lebard and Matyushov 2009), enzymatic reaction efficiency determination (Jackson et al. 2009; Bhabha et al. 2011), mutations observed in protein domain families (Leo-Macias et al. 2005; Echave 2008; Echave and Fernandez 2009; Velazquez-Muriel et al. 2009), and the divergence of duplicate gene functions (Glembo et al. 2012).

Despite extensive evidence of the critical role of protein dynamics in function, static (motionless) structures are primarily used in molecular biology and evolution, where individual structural residues are frequently categorized into structural motifs (e.g., α-helices, β-strands, and loops), functional attributes (e.g., binding and interacting residues) (Jordan et al. 2010), and estimating accessible surface areas (ASA) and residue–residue interaction information, among others measures (Cheng et al. 2008; Adzhubei et al. 2010; Martin et al. 2011). However, the proper function of the cell is maintained through the interactions of proteins in a crowded environment, where each protein maintains its function through structural dynamics within a broad range of scales, from atomic fluctuations and side-chain rotations to collective domain movements. Moreover, mutational changes in a given residue position will have a larger impact on protein dynamics (both locally and globally) as compared with structural changes, which has been observed to produce functional effects (Gunasekaran, Ma, and Nussinov 2004; Potapov et al. 2009; Kellogg et al. 2011; Ma et al. 2011; Glembo et al. 2012). Therefore, a need exists for quantitative measures that capture the contribution of each amino acid position to functionally related structure dynamics. Such a position-specific dynamics measure will then allow for a comprehensive evaluation of the importance of dynamic flexibility of protein positions to their biological function(s). Moreover, with this position-specific dynamics measure, we will be able to incorporate structural dynamics into genomic analysis and provide general evidence for the critical role of protein dynamics in shaping the biological functions through a proteome-wide analysis using a large number of proteins of any species.

We describe a novel metric called the dynamic flexibility index (*dfi*) that measures the dynamic response of each specific position, when a perturbation such as a random Brownian kick is introduced to a protein. This perturbation indeed mimics the nature, since a protein is exposed to many random forces as a first order approximation in a crowded cell while interacting with other proteins or ligands. Therefore, under the hypothesis that there is an underlying dynamics (i.e., fluctuation profile) for the three-dimensional (3-D) structure of a protein, which is crucial for the function, our metric *dfi* quantifies the contribution of each position to this functional dynamics and is designed to capture the key residues mediating the function through the residue interaction dynamics. To explicitly evaluate the role of structural dynamics in proper biological functioning, we rigorously analyze various biological and functional properties of *dfi* using a diverse collection of human proteins with known experimental structures.

## Methods

### Protein data set

We find that 100 proteins analyzed in ref. (Kumar et al. 2009) have 3-D structures in the protein databank (Bernstein et al. 1977), such that there is >90% sequence identity between the reference sequence and the known protein structures with a > 90% sequence coverage when using BLAST (see Data Set in Data S1). We obtained a representative set of 100 protein structures having 39,813 residues, with non-redundant positions of 792 disease-associated alleles and 788 neutral alleles.

### The formulation of DFI

We used the Perturbation Response Scanning (PRS) technique that combines the Elastic Network Model (ENM) and Linear Response Theory (LRT) (Atilgan et al. 2001; Ikeguchi et al. 2005; Atilgan and Atilgan 2009; Atilgan et al. 2010). In ENM, a protein structure is viewed as a 3-D elastic network and all residue pairs are subject to a uniform, single-parameter harmonic potential if they are located within an interaction range, or cutoff distance (Tirion 1996; Hinsen 1998; Atilgan et al. 2001). In ENMs, the expansion of the potential near the equilibrium state can be written in compact notation as 

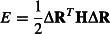
1

Here, Δ***R*** is the 3N-dimensional vector of fluctuations of all residues, and ***H*** is the Hessian, a 3N × 3N matrix composed of the second derivatives of the harmonic potential with respect to the components of the positions vectors of length *N*. In this study, we weighted the interaction strength between all residue pairs by using the inverse of the square distance of their separation (Lin et al. 2008; Yang et al. 2009), rather than using arbitrary cutoff distances (Hinsen 1998; Yang et al. 2009).

**Figure 1:**
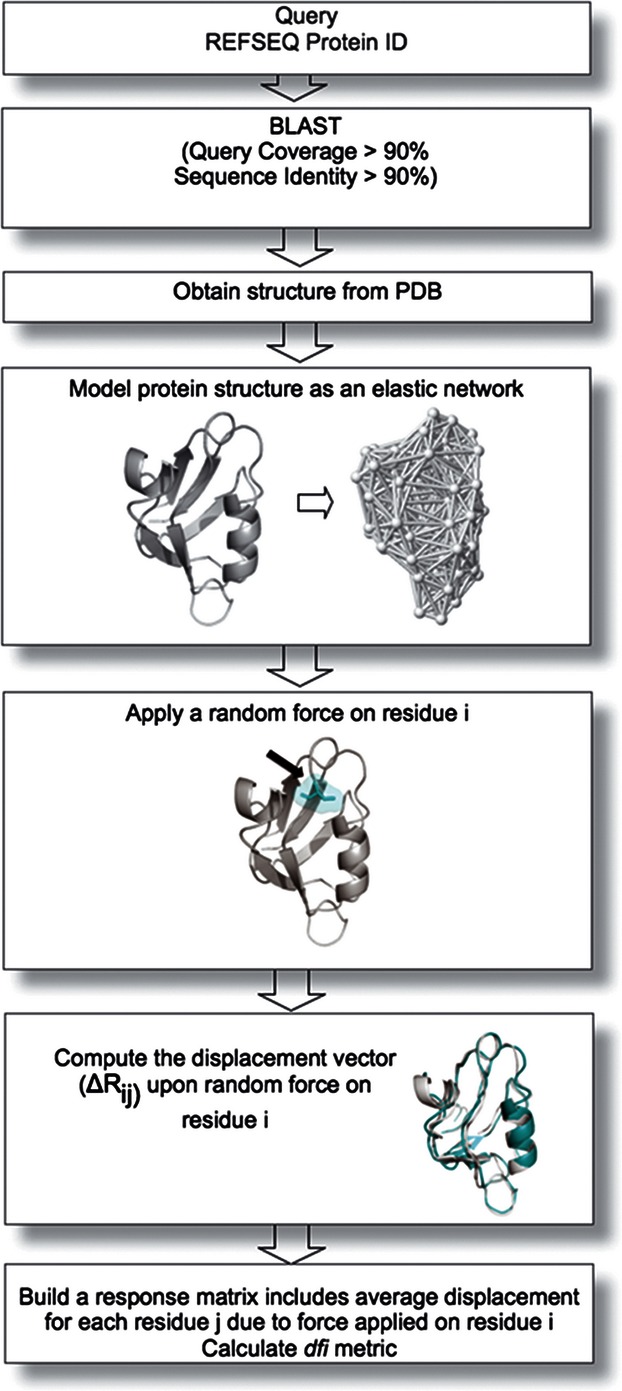
The schematic diagram of the method followed for structural dynamics analysis of each protein. We identify a three-dimensional (3-D) structure for each protein sequence in the data set (Kumar et al. 2009) through a BLAST search using protein data bank (PDB) (Bernstein et al. 1977). In this search, the sequence coverage and the sequence identity between the reference sequence query and the known protein structures is set to >90% and >90%, respectively. The identified 3-D experimental structures from PDB are then used for the Perturbation Response Scanning (PRS) model to predict the dynamic flexibility index (%*dfi*) for each residue position.

After obtaining ***H***, we sequentially exert directed random unit forces on single-residues along the chain of the structure and record the resulting relative displacement of all residues using linear response theory (LRT) as 

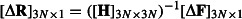
2
where the Δ***F*** vector contatins the components of the externally applied random unit force vectors (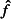) on the selected residues and ***H***^***-1***^ is the inverse of Hessian matrix. To minimize the effects of randomness, the perturbation procedure is performed 10 times to ensure the force applied is isotropic with the zero angular average (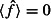), and then the response vector ΔR^i^_j_ is averaged. Then, we build a perturbation response matrix that includes the average displacement for each residue *j* due to the random force applied on residue *i*. That is, 

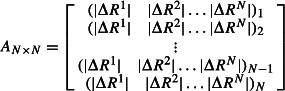
3
where 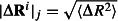 is the magnitude of positional displacements for residue *j* in response to a perturbation at residue *after averaging out* the response vector ΔR^i^_j_ *over the ten different random directional unit forces*. The rows of this matrix show the response fluctuation profile of each position upon perturbation of a specific residue. On the other hand, the columns of the matrix represent the average displacement of a specific residue from its mean position, when other residues are perturbed one at time along the chain. After generating perturbation response matrices upon exerting an external force at several directions, we calculate the average of the total amount of displacement for residue *j* (i.e., mean square fluctuation) induced by perturbations placed on the rest of the residues in the chain, 

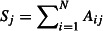
4
where *A*_ij_ is response fluctuation profile of residue *j* upon perturbation residue *i*. Then, we define a relative metric called the dynamical flexibility index (*dfi*) for each residue 

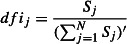
5
where *S*_j_ values are normalized by the average of the total amount of displacement of the residue *i* over the average displacement of all residues The outlines of our approach is shown in Fig. 1. In addition to the coarse-grained approach, we also use all-atom replica exchange molecular dynamics (MD) trajectories to estimate the root-mean squared fluctuation (*rmsf*) of the atoms around their original positions for computing *dfi* (See Data S1 and Figure S1).

### Structural and evolutionary parameters

We estimate the absolute evolutionary rate at each site by using a previously described method (Kumar et al. 2009), which computes the number of amino acid substitutions in a given phylogeny following the parsimony algorithm (Fitch 1971). The evolutionary rate of amino acid change across species is then the number of amino acid substitutions divided by the total time elapse in the tree. Evolutionary rates are in the units of substitutions per amino acid per billion years (Byrs) and are based on protein sequence alignments of 46 species available from the University of California-Santa Cruz (UCSC) resource (Kent et al. 2002). For structural parameters, we computed ASA for each residue, ASA by using Surface Racer program (Tsodikov et al. 2002) with a probe radius of 1.4 A˚, corresponding to the size of a water molecule. The change in protein folding free energy (ΔΔG) upon mutation was estimated using the FoldX method (Guerois et al. 2002), where the energies of the wild type experimental structures are optimized using the ‘Repair protein data bank (PDB)’ command and then modeled individual mutations using ‘BuildModel’ command to obtain ΔΔG.

## Results

### Estimation of DFI

For estimating *dfi*, we first construct a 3-D elastic network for the tertiary protein structure, in which the interacting Cα atomic coordinates of each residue are linked with an elastic spring. The ENM is chosen because it has been found to capture the conformational protein dynamics and predict functionally important residues (Tirion 1996; Hinsen 1998; Atilgan et al. 2001; Tama and Sanejouand 2001; Zheng et al. 2006, 2007; Kurkcuoglu et al. 2009; Bahar et al. 2010a; Bahar et al. 2010b). On this 3-D ENM, we apply a random Brownian kick to a given residue in the chain, which perturbs the residue interaction network of the protein beyond fluctuations inherent in the system at equilibrium and elicits responses from all other residues in the structure. This procedure indeed mimics the natural process of interactions in the cell as a first order approximation, since an approaching ligand applies forces on the receptor protein, inducing conformational change. Through the PRS method, we compute the fluctuation response of residue *j*, ΔR^i^_j_, *both in direction and magnitude* upon perturbation. In short, the response fluctuation profile, ΔR^i^_j_, gives deviation of the residue j from its mean position in x, y, and z direction upon perturbing residue *i*.

The PRS couples ENM with LRT (Atilgan et al. 2001; Ikeguchi et al. 2005; Atilgan and Atilgan 2009; Atilgan et al. 2010; Gerek and Ozkan 2011). The PRS has already proven to be a powerful approach to capture conformational changes upon binding (Atilgan et al. 2010) and has been useful for identifying key residues that mediate long-range communication and finding allosteric pathways (Gerek and Ozkan 2011). The magnitude of response by residue *j* due to a Brownian kick at residue *i* is given by the mean square fluctuation |*Δ***R**^*i*^|_*j*_ (see Methods for details). The mean square fluctuation is estimated for every residue's response to Brownian kicks at all other residues. Then, we estimate *dfi* of residue *j* using the following equation (See Fig. 1 for the method algorithm).

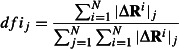
6

As defined, *dfi* is a relative value, indicative of being higher or lower than the average response observed at any position in a protein structure. It measures the individual position's resilience to perturbations within given the 3-D structure as it occurs through binding or catalytic activity or due to mutations. The residue positions with very low *dfi* indicate dynamic stability, as they can absorb and transfer the perturbation throughout the chain in a dynamic cascade fashion. Thus, they will often be the hinge parts of the protein that control the motion like joints in a skeleton. On the other hand, sites with very high *dfi* are prone to perturbations to the amino acid chain. They are structurally flexible sites. Overall, the dfi measures the significance of each position's contribution to the functionally important dynamics.

Above, we have described a coarse-grained approach for calculating *dfi*, which uses Cα coordinates for predicting residue fluctuations. An alternative is to employ an all-atom MD simulation to estimate the *rmsf* of the atoms around their original positions (i.e., covariance matrix) for computing *dfi*. We compared *dfi* values using the coarse-grained approach with those obtained from all-atom replica exchange MD (REMD) trajectories (See Data S1 for details). These two estimates show high correlations (Figure S1). However, the MD approach is computationally intensive and not always feasible. For instance, the coarse-grained approach takes less than a minute on a dual core computer to compute *dfi* for a protein of 243 residues, as compared to 260 CPU hours needed for MD simulations [5 ns run; REMD with Amber force field (Ozkan et al. 2007)]. This is a four order of magnitude difference in time requirements. In addition, MD simulations failed to converge for longer proteins, even after thousands of CPU hours. Therefore, we have used only the coarse-grained approach (PRS) in the rest of the analyses.

### Importance of dynamic flexibility of residues in biological phenotypes

In nature, *de novo* mutations are occurring randomly and are constantly subjected to natural selection. Many mutations that significantly impact organismal fitness (owing to the disruption of protein function) manifest themselves in the form of diseases in populations, whereas mutations with small or insignificant fitness effects are found as polymorphisms (Kumar et al. 2009, 2011). Abundant availability of these two types of variations enables us to directly assess the relationship between the *dfi* and the biological phenotype. If there is a strong dependence of the latter on the *dfi,* then we would expect to reject the null hypothesis that disease-associated variants are distributed uniformly in residues with low and high dynamic flexibilities.

We used Mendelian disease-associated variants to test this hypothesis, because they are monogenic diseases where individual amino acid mutations are strongly linked with the genetic disease (Kumar et al. 2009, 2011). We retrieved experimentally derived structures of 100 proteins through a BLAST search of the data set that contains at least one variant [disease-associated or neutral per sequence (Kumar et al. 2009)]. As mentioned above, the *dfi* is a protein specific measure for individual 3-D structure. Therefore, its use in collective analysis of residue positions across different protein structures requires normalization. This is achieved by expressing the *dfi* value of a residue position as a percentile rank of that residue in a sorted array of all *dfi* values in the given protein (%*dfi*).

**Figure 2:**
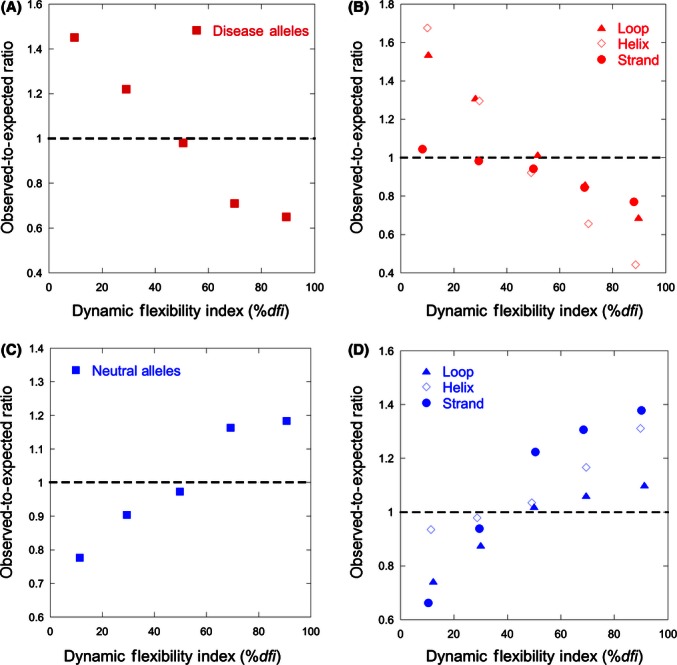
The relationship of the observed-to-expected numbers of disease variants found in the human population from 100 proteins for all disease-associated variants (A), disease-associated variants in different secondary structural motifs (B), all polymorphisms (C), and polymorphisms in different secondary structural motifs (D). The expected number of variants at the amino acid positions for a given dynamic flexibility index (%*dfi)* category, *i*, is computed as E_i_ = (n_i_/N) × M, where n_i_ is the number of amino acid positions belonging to the i^th^ category, N is the total number of amino acid positions, and M is the total number of disease-associated (or neutral) variants used in this analysis. A chi-squared (χ^2^) test is applied to evaluate the significance of the deviations of the observed values from the expected values. In all cases, the null hypothesis is rejected with a *P* << 0.001.

For 792 (Mendelian) disease-associated variants, we estimated the expected numbers of positions that will contain variants in five categories: *dfi* < 20%, 20% ≤ *dfi* < 40%, 40% ≤ *dfi* < 60%, 60% ≤ *dfi* < 80%, *dfi* ≥ 80%. Under the null hypothesis of no effect, the ratio of the expected and observed numbers of residue positions hosting disease-associated variants should be close to 1.0 for each category, which is rejected (*P* << 0.05; Fig. 2A). Residues with the lowest *dfi* show the highest enrichment of disease-associated variants (ratio = 1.45), whereas those with the highest dynamic flexibility show a major deficit of these variants (ratio = 0.65). Residues with intermediate %*dfi* show intermediate effects (Fig. 2A). The result is robust to the number of %*dfi* categories used and holds true even when we analyze disease-associated variants separately for helices, loops, and sheets (Fig. 2B).

These expected to observed numbers indicate that the robustness of residues with the lowest *dfi* values, which are not otherwise affected by long and short-range protein perturbations, is disrupted the most by disease-associated mutations. It also predicts that population variants with no known disease-association (neutral variants) will be less frequent in low *dfi* residues. This prediction is also verified with an analysis of 788 neutral variants, which shows a large overabundance at residues with high %*dfi* (Fig. 2C). This pattern is also observed in an analysis of neutral variants in different secondary structure classes (Fig. 2D). Thus, positions with high *dfi* values accommodate amino acid variations more frequently.

Overall, the above analysis indicates that the need to maintain robustness of residues from a structural dynamics perspective is continuously shaping the protein variation present in a population. By the *dfi* analysis on a large number of disease and neutral variants obtained from the human proteome, we establish the importance of structural dynamics to biological function independent of other biochemical attributes, because *dfi* is solely based on protein dynamics considerations.

### Dynamic flexibility of residues involved in catalytic and binding functions

**Figure 3:**
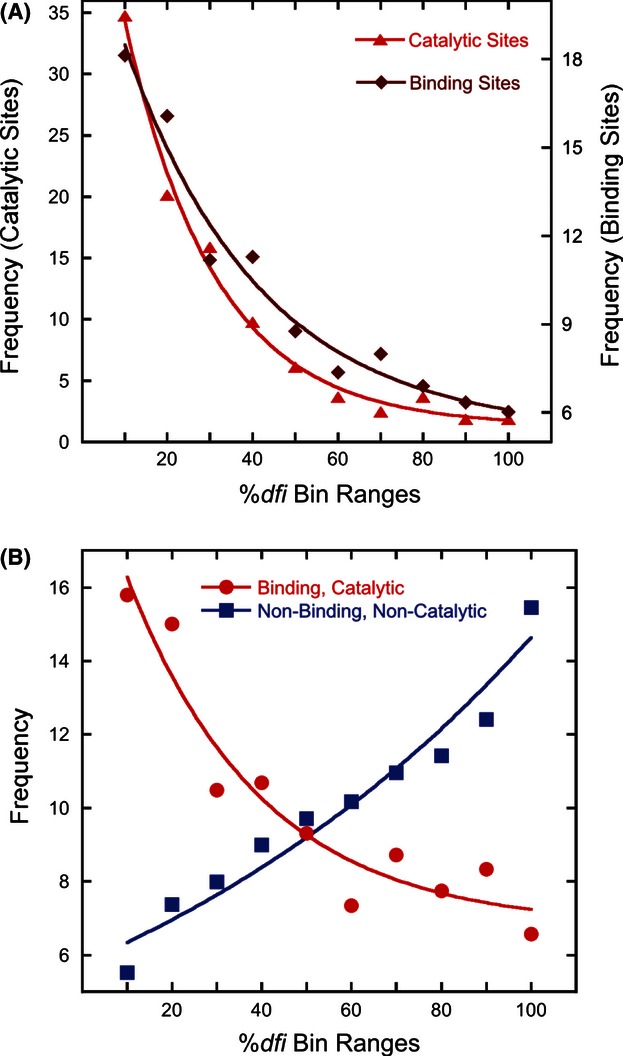
Dynamic flexibility profiles of the residues involved in catalytic and binding activities. (A) Frequency distributions of dynamic flexibility index (%*dfi)* are shown for all residues involved in catalytic (164; red triangles) and binding (1938, maroon diamonds) activities. (B) Frequency distributions of %*dfi* for residues in loops involved in catalytic and binding activities (red circles), which show a trend opposite from that seen for all other loop residues (blue squares).

We also examined the distribution of %*dfi* values for structural residues involved in binding and catalytic functions in the 100 proteins analyzed above. Using the PDBsum server (Laskowski et al. 1997; Laskowski 2009) and Catalytic Site Atlas (CSA) (Laskowski et al. 1997; Laskowski 2009), we generated a data set containing 1874 residues that interact with ligands or small compounds (76 proteins), 96 residues involved in catalytic activities (34 proteins), and 68 residues that are annotated to have both binding and catalytic activities (31 proteins). The residues with catalytic functions generally have lower dynamic flexibility (Fig. 3A), with over 50% of the catalytic residues showing %*dfi* ≤ 25%. That is, they tend to be among the most dynamically robust residues. This is biologically reasonable because catalytic sites are often co-localized with inflexible hinge residues (Yang and Bahar 2005) or buried within the protein core due to their special enzymatic activity (Bartlett et al. 2002), and therefore, they should be also more robust to perturbations and exhibit low *dfi* profiles. On the other hand, the binding residues exhibit a higher degree of flexibility than catalytic residues (Fig. 3A), which is consistent with their need to accommodate binding-induced conformational change.

In our data set, almost half of the residues involved in catalytic and binding activities occurred in the loops (1020 out of 2038), which are the most flexible regions in a protein structure. This prompted us to evaluate the *dfi* values of these loop residues involved in catalytic and binding activities. We find that the residues with such activities show opposite %*dfi* distributions as compared with all other residues located in loops (Fig. 3B). This means that even though loops generally harbor residues with higher %*dfi* values (due to their propensity to be easily displaced), the functionally critical positions even within loops show a tendency to be dynamically less flexible than other positions.

### Evolvability of positions with low and high dynamic flexibilities

The above analyses establish the functional implications of the dfi through the effects of positions afflicted with disease-associated and neutral variation in proteins and residues involved in catalytic and binding activities. However, those analyses only permit an examination of the properties of only a small fraction of 39,813 total residues present in 100 proteins analyzed. To extend the analysis to all the positions in the proteins, we examined the relationship of %*dfi* and position-specific rate of evolutionary change obtained from a multispecies sequence analysis, with the position-specific evolutionary rates serving as a proxy for functional importance. In this case, dynamically more important positions will be under stronger natural selection over time, which will permit fewer amino acid substitutions at those positions. (Of course, many other functional factors will influence the evolutionary conservation, including the catalytic activity, roles of charge and hydrophobic residues, structural stabilization needs, and post-translational modifications.)

**Figure 4:**
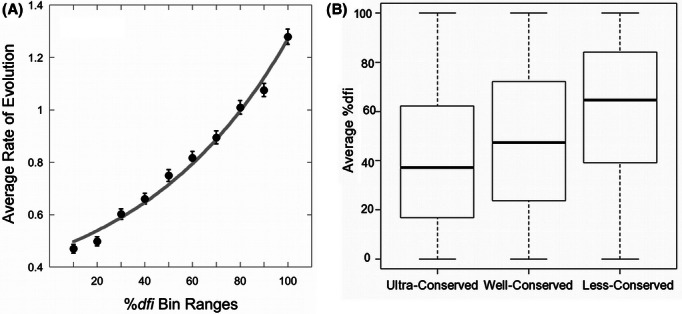
Relationships of residue evolutionary rates and dynamic flexibilities. (A) Average evolutionary rate of change of residues with increasing dynamic flexibility (%*dfi*) in a sliding window. The correlation between the average evolutionary rate and the average %*dfi* is 0.85. (B) Boxplot of the average %*dfi* distributions on ultra-conserved, well-conserved, and less conserved residues. The amino acid substitution rates (*r*) for these categories are *r* = 0, 0 < *r* ≤ 1, *r* > 1, respectively. Box plots show median, upper, and lower quartiles, and whiskers show maximum and minimum values.

Therefore, we estimated the rate of amino acid change per site per Byrs (*r*) for all the positions in 100 proteins (see Methods) and explored its relationship with dynamic flexibility (%*dfi*). There is a direct positive relationship between %*dfi* and *r* (Fig. 4A; correlation = 0.85). The positions with lower *dfi* values are the most constrained evolutionarily, and the most highly conserved positions show the lowest *dfi* profiles, on average (Fig. 4B; correlation = 0.73). These patterns are also supported by experimental observations of lower structural mobility of conserved residues in nuclear magnetic resonance (NMR) analysis (Mittermaier et al. 2003) and in theoretical analysis of fluctuation profiles (Adzhubei et al. 2010; Liu and Bahar 2010).

## Discussion

We have described a novel quantitative measure of dynamic flexibility (*dfi*) of individual residues that use a (3-D) elastic network for the tertiary protein structure. Three different types of analyses involving functionally critical positions, population variations, and interspecific substitutions produce concordant patterns, which establish that the preservation of dynamic properties of residues in a protein structure is critical for maintaining the protein/biological function.

Protein structural metrics such as ASA is also frequently used to assess the functional importance of individual residues (Franzosa and Xia 2009; Wilke and Drummond 2010; Toth-Petroczy and Tawfik 2011). Interestingly, we found that the ASA difference between functionally critical and non-critical sites involved in catalytic or binding activity in our data set is not statistically significant (*P* > 0.08), whereas the difference of *dfi* between these sites is highly significant with *P* < 0.00001 as also shown for residues located in loops (Fig. 3B). Contrary to the general observation that disease-associated sites have low ASA values (David et al. 2012; Wei et al. 2013), we found that specific disease sites in several proteins in our data set show high ASA values. Strikingly, for all of these cases, our *dfi* analysis shows that these sites exhibit low *dfi* values, indicating that they are prone to diseases as a few examples are shown in Figure S2. Overall, these findings suggest that the metrics based on structural dynamics have the ability to discriminate functionally crucial positions beyond the static structural features.

**Figure 5:**
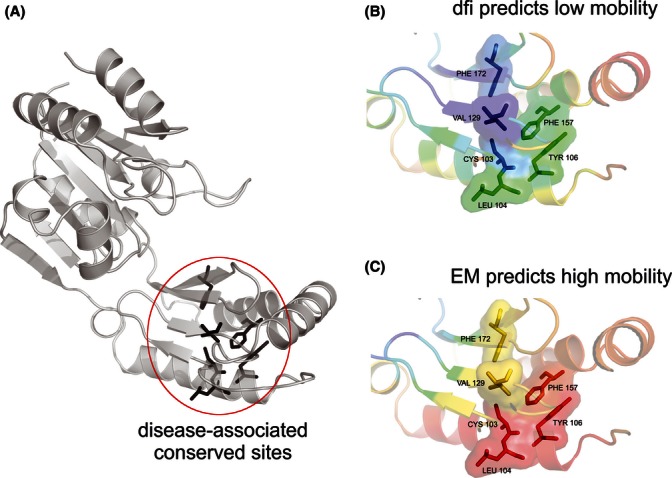
The ribbon diagrams of human phosphomannomutase 2 (NP_000294) with respect to (A) dynamic flexibility index, %*dfi* and (B) effective mobility (%EM). Each structure is colored within a spectrum of red–yellow–green–cyan–blue, where red shows the highest and blue the lowest values of %*dfi and %EM*. All disease variants shown as surface are slow evolving positions where evolutionary rate, *r* ≤ 1. The average %EM of disease variants is high (%54), but the average %*dfi* is quite low with a value of 24%. Similar observations were found in 4-Sulfatase (NP_000037) and human protoporphyrinogen IX oxidase (NP_000300), respectively.

The correlation between protein dynamics in terms of effective mobility (EM) and evolutionary conservation has been reported for some enzymes recently (Liu and Bahar 2012), which is consistent with our findings (Fig. 4 above). This is because EM is a special case of *dfi* where a one-dimensional ENM approach (Bahar et al. 1997) is used to primarily capture correlations between the fluctuations at equilibrium using the slowest modes of motion governed by the 3-D structure. This means that EM ignores the effect of perturbations when the structure is displaced out of equilibrium as compared to *dfi*, which is crucial to detecting the underlying features of the energy landscape. For example, the functional regulations in small domain proteins frequently arise through changes in the residue-dynamics rather than large domain movements (Dima and Thirumalai 2006; Smock and Gierasch 2009; Gerek and Ozkan 2011). Our approach automatically considers multiple normal modes (i.e., specific frequency of motion) and distinct higher frequency modes that may contribute to functional dynamics. This is likely the reason for the observation that the use of EM for our data did not distinguish between residues harboring disease-associated and neutral variations at the conserved positions (see Methods). These positions show the highest propensity of containing disease-associated variations (Miller and Kumar 2001; Kumar et al. 2011), and *dfi* is able to discriminate between disease-associated and neutral variations at these positions (*P* << 0.0001; based on *t*-test with unequal variance). One example of such a position is shown in Fig. 5 for the phosphomannomutase 2 protein. Here, the average EM value of the disease variants harboring at slow evolving positions is very high (54%), which disagrees with the common observation of the low mobility profile of disease-associate variants. Conversely, the average *dfi* is rather low (24%), in agreement with our previous finding that it can differentiate disease variants.

The crystallographic B-factor, which describes the attenuation of X-ray scattering caused by thermal motion, has been previously used in the prediction of functionally damaging variation (Chasman and Adams 2001; Ramensky et al. 2002; Adzhubei et al. 2010). To compare our metric *dfi* with the B-factor, we use a subset of 37 proteins (615 disease-associated and 265 neutral variants), because for the accuracy of the B-factors we need crystallographic proteins with better than 3 Å resolution available in our data set. In this small subset, we found that %*dfi* shows a relatively higher difference between disease-associated and neutral variants than that shown by B-factors (40% higher with *P* < 0.001) Besides its higher predictive power in disease-associated variants, *dfi* can be applied more broadly to low-resolution crystal structures, homology models, and proteins resolved using NMR experiments.

The change in stability (ΔΔG) is also used to evaluate function-impacting propensity of mutations (Yue et al. 2005; Cline and Karchin 2010; Jordan et al. 2010). In our analysis, however, ΔΔG also did not provide discrimination, as it was positive as often as it was negative (53% vs 47%) for disease-associated variants, whereas the %*dfi* of disease variation harboring positions was much lower than expected (Fig. 1). This observation is consistent with the recent findings that ΔΔG does not have strong discrimination power (Potapov et al. 2009) unless the protein conformational sampling upon mutations are considered (Kellogg et al. 2011; Juritz et al. 2012; Wickstrom et al. 2012).

In conclusion, we have taken a phylomedicine approach to evaluate the usefulness of the newly proposed matric (*dfi*) and shown that it holds promise for us in discriminating between disease-associated and neutral variation. In the future, we envision that it will complement existing structural matrices and be used alongside evolutionary and functional information in building more sophisticated predictive models to forecast the biological severity of new mutations that are being discovered at a fast pace because of personal exome sequencing in fundamental research and clinical applications (Kumar et al. 2011).

## Supporting Information

Data S1 Validation of coarse-grained approach by using all atom Molecular Dynamics.
